# Assessment of the Spoilage Microbiota during Refrigerated (4 °C) Vacuum-Packed Storage of Fresh Greek Anthotyros Whey Cheese without or with a Crude Enterocin A-B-P-Containing Extract

**DOI:** 10.3390/foods10122946

**Published:** 2021-11-30

**Authors:** Nikoletta Sameli, Eleni Sioziou, Loulouda Bosnea, Athanasia Kakouri, John Samelis

**Affiliations:** Dairy Research Department, Institute of Technology of Agricultural Products, Hellenic Agricultural Organization “DIMITRA”, Katsikas, 45221 Ioannina, Greece; nikol.sameli@gmail.com (N.S.); eleni.sioziou@gmail.com (E.S.); loulouda.bosnea@gmail.com (L.B.); kakouriathanasia@yahoo.gr (A.K.)

**Keywords:** whey cheese, Anthotyros, *Leuconostoc mesenteroides*, *Carnobacterium maltaromaticum*, *Hafnia*, *Serratia*, *Pseudomonas*

## Abstract

Although fresh whey cheeses are prone to rapid deterioration, mainly by psychrotrophic Gram-negative bacteria and lactic acid bacteria (LAB), data on the specific spoilage species in traditional Greek whey cheeses are scarce. Therefore, this study quantified growth and characterized the primary spoilage bacteria in fresh Anthotyros whey cheeses stored at 4 °C in a vacuum for 40 days, without or with an added 5% (*v*/*w*) of an enterocin A-B-P crude extract (CEntE). Psychrotrophic *Pseudomonas* spp., *Aeromonas* spp., *Hafnia* spp. and *Serratia* spp. grew faster than LAB during early storage. However, LAB outgrew the Gram-negative bacteria and prevailed by mid to late storage in all cheese batches, causing a strong or milder batch-dependent natural acidification. Two major non-slime-producing and two minor biotypes of *Leuconostoc*-like bacteria, all identified as *Leuconostoc mesenteroides* by 16S rRNA sequencing, dominated the LAB association (76.7%), which also included four subdominant *Carnobacterium maltaromaticum* biotypes (10.9%), one *Leuconostoc lactis* biotype (3.3%) and few *Lactococcus* (1.6%), mesophilic *Lactobacillus* (0.8%) and *Enterococcus* (0.8%). Growth and distribution of LAB and Gram-negative species were strongly batch-dependent and plant-dependent. The CEntE neither retarded growth nor altered the whey cheese spoilage association but enhanced LAB growth and the declines of Gram-negative bacteria by late storage.

## 1. Introduction

Whey cheeses represent a specific category of fresh cheeses produced from the remaining whey following the manufacture of typical rennin-coagulated cheeses [1,2]. Their manufacture is based on the denaturation and coagulation of water-soluble milk proteins, mainly α-lactalbumin and β-lactoglobulin, of the whey following heating at high temperatures (>80–95 °C). The curd is most typically collected as a surface pellicle from the coagulating whey system. To enrich whey cheeses in protein and fat, milk or milk fat may be added at different concentrations to the whey before heating [1,3]. Due to the heating of the whey, the natural microbiota is inactivated, starter cultures are not applicable and thereby whey cheeses are practically free of microorganisms after manufacture, provided that the hygienic conditions employed are strict. However, handling of the fresh curd post-heating results in cross-contamination with a large variety of spoilage and potentially pathogenic bacteria, including mainly psychrotrophic spoilage LAB, *Enterobacteriaceae* and *Pseudomonadaceae* [1,4,5]. Therefore, fresh whey cheeses are highly perishable dairy products due to their high pH (>6.0–6.8) and moisture content (>60–80%): they are prone to rapid bacterial deterioration, especially at abusive (>7–12 °C) storage temperatures [1,4,5,6,7].They are also an excellent substrate for dairy pathogens, especially for *Listeria monocytogenes*, that may outgrow in the absence of a protective microbiota [1,3,7,8,9].

Globally, Ricotta cheese, originally from Italy, is the most popular and most investigated fresh whey cheese variety, particularly with studies pertaining to various hurdle approaches to retard growth and monitor the composition of the spoilage microbiota to extend the shelf-life and to inhibit pathogens to increase safety [5,10,11,12,13,14]. A large variety of whey cheeses that are consumed fresh or after natural drying/ripening are traditionally produced in the Mediterranean basin, particularly in eastern Mediterranean countries [1,2]. In Greece, the most popular and most investigated whey cheeses of major economic and nutritional importance are Myzithra, Anthotyros and Manouri [3,9,15,16,17]. They are produced at both industrial and artisan scales from the whey of Feta cheese or cooked hard cheeses (e.g., Kefalotyri, Graviera) [16,17]. A maximum moisture content of 70, 70 and 60% and a minimum fat content in dry matter of 50, 65 and 70% are permitted for fresh Myzithra, Anthotyros and Manouri, respectively [3,16]. Hence, traditional fresh Greek whey cheeses stored aerobically spoil rapidly by psychrotrophic bacteria during refrigerated storage [15,18,19,20]. Sometimes, the retail shelf-life of Greek whey cheeses stored in air is limited to 5–7 days, while vacuum packaging (VP) and modified atmosphere packaging (MAP) have been shown to extend their shelf life by 10 to 20 days, depending on the cheese variety and several other interfering factors, such as the levels and types of microbial post-process contaminants associated with the plant hygiene and potential temperature fluctuations during retail distribution and storage [3,17,21,22,23].

Overall, refrigeration alone, or combined with VP or MAP, cannot prevent fresh whey cheese spoilage, although the oxygen decrease and carbon dioxide increase in VP or MAP shifts the natural cheese spoilage association from mostly aerobic Gram-negative to anaerobic Gram-positive bacteria, primarily lactic acid bacteria (LAB) with a milder spoilage potential [1,4,5,6,21,24,25,26]. However, several microaerophilic non-LAB spoilage bacteria, such as psychrotrophic *Enterobacteriaceae*, and pathogen contaminants, particularly *L. monocytogenes*, retain high growth potential under VP or MAP cold storage conditions [3,27], which necessitates the in-package application of additional hurdle measures to increase the shelf-life and safety of fresh (Greek) whey cheeses [11,12,23].

Biopreservation by means of natural antimicrobials or protective LAB cultures is one of the most attractive and consumer-friendly methods to increase the shelf-life and safety of fresh whey cheeses during retail distribution and storage [14,23,28,29]. However, nisin-producing (Nis+) *Lactococcus lactis* or other bacteriocinogenic (Bac+) dairy starter LAB strains able to produce sufficient amounts of active bacteriocin (nisin) molecules in situ in fermenting cheese milks are not well-applicable in fresh whey cheeses because they grow poorly under refrigeration. Conversely, at abusive (>7–12 °C) temperatures *L. lactis* and other Bac+ mesophilic dairy LAB strains affect the desirable sensory quality characteristics of high-pH ready-to-eat (RTE) whey cheeses by a simultaneous high production of organic acids during retail storage. To overcome low-pH defects, bioprotective strains of non-aciduric LAB species/genera or Nis+/Bac+ mutant lactose-negative dairy LAB strains may be applied [28,29,30]. An alternate option is the addition of commercial nisin (Nisaplin^®^), pediocins or various crude LAB bacteriocin concentrates in fresh whey cheeses [3,10,28,30]. However, Nisaplin^®^ added as an antilisterialbiopreservative to the whey (100 or 500 IU/g) before heating, or to the fresh cheese curd (500 IU/g) before VP, reversed the natural predominant spoilage microbiota of traditional Anthotyros from Gram-positive LAB in the control cheeses without nisin to Gram-negative bacteria during storage at 4 °C for up to 45 days, particularly in the most effective Nisaplin^®^ treatments [3].

A similar approach is the addition in the fresh (whey) cheeses of enterocin-producing (Ent+) dairy enterococci. The best candidates are selected safe Ent+ strains of *Enterococcus faecium*, *Enterococcus durans* and *Enterococcus faecalis* without β-hemolytic activity, virulence genes, vancomycin resistance genes and other undesirable traits. An increasing number of promising experimental applications of Ent+ strains or enterocin preparations in dairy foods, particularly active against *L. monocytogenes*, are summarized in recent reviews [28,30,31], including a few studies on fresh whey cheeses [32]. Most enterococci are naturally non-aciduric LAB, which is advantageous for Ent+ strain applications in fresh RTE whey cheeses. Moreover, apart from *L. monocytogenes*, the antimicrobial spectrum of several enterocins and/or Ent+ strains has been reported to include a wide range of Gram-positive sporoforming as well as Gram-negative bacteria [28,29,30,31,33]. On the other hand, most enterococci promote negligible or poor growth at refrigeration temperatures [33], which is against outnumbering other psychrotrophic spoilage or pathogenic bacteria with sufficient in situ enterocin production in fresh cold-stored whey cheeses [14]. In this aspect, the addition of purified, semi-purified or even crude enterocin extracts [28,30,34] may be more effective than the direct addition of viable Ent+ *Enterococcus* spp. for controlling the growth of native spoilage bacteria and *L. monocytogenes* infresh whey cheeses.

Although the spoilage microbiota of Anthotyros, Myzithra and Manouri has been studied broadly, i.e., in terms of quantifying different bacterial groups and yeasts during storage in air, VP or MAP [17,18,19,21,22,23], limited micro-ecological data on the specific spoilage LAB and non-LAB species [18,20], and no application studies of enterocins, Ent+ enterococci or other bioprotective LAB cultures, exist. Therefore, this study was undertaken to monitor the evolution of spoilage microbiota and to identify specific spoilage species during refrigerated storage of fresh Anthotyros whey cheeses in VP. Additionally, the effects of a crude enterocin A-B-P-containing extract on the growth and the species composition of the Anthotyros spoilage association were determined.

## 2. Materials and Methods

### 2.1. Preparation of the Crude Enterocin A-B-P Biopreservative Extract

Three autochthonous single (Ent+) or multiple (m-Ent+) enterocin-producing strain biotypes of *E. faecium*, originally isolated from traditional Greek Graviera cheese [35], recently genotyped, characterized for their enterocin-mediated activity in culture broth media and skim milk [36] and evaluated for their safety [37], were used. They were the EntA+ *E. faecium* KE64 (GenBank accession no. MW644963), the m-Ent+ (*entA-entB-entP*) *E. faecium* KE82 (MW644969) and the m-Ent+ (*entA-entB-entP*) *E. faecium* KE118 (not yet deposited in GenBank) [37]. The strains were activated from their frozen (−30 °C) stock state in de Man, Rogosa, Sharpe (MRS) broth (Lab M, Heywood, UK), with 20% glycerol, by adding 0.1 mL of each stock culture in 10 mL MRS broth incubated at 30 °C for 24 h and then subcultured twice, as above. For the preparation of the mixed enterocin extract, 0.5 mL of a fresh (24h) culture of each strain was added to 50 mL of MRS broth, incubated at 30 °C for 48 h, followed by centrifugation (3200× *g* for 15 min) to remove the cells. The supernatants were filter-sterilized without preceding pH adjustment and combined in a pre-sterilized 250mL Duran flask. This 150mL cell-free supernatant (CFS) composite that contained enterocin A (secreted by all three strains) plus enterocin B and P (secreted by KE82 and KE118) was designated the crude enterocin A-B-P-extract (CEntE); it was stored at −30 °C until use in the whey cheese experiments.

### 2.2. Commercial Anthotyros Whey Cheese Samples

Eight retail vacuum packed (VP) samples of fresh, traditionally manufactured Anthotyros whey cheese were obtained from two commercial semi-industrial dairy plants located in Epirus, Greece. Specifically, the eight VP samples represented four independent whey cheese batches: A, B, C and D.Two individual VP samples were taken from each batch; each VP contained 500 g of fresh 24-h-old cheese. Batches A and B were products of the traditional dairy Pappas Bros. (Skarfi E.P.E., Filippiada, Epirus), our collaborator SME in the BIO TRUST project, while batches C and D were fresh retail Anthotyros cheese products of another traditional dairy located near Ioannina. All VP samples were transported to the microbiology laboratory of the Dairy Research Department at Ioannina in insulated ice boxes and used in the experiments within one hour after transportation.

### 2.3. Enterocin Addition and Cheese Storage and Sampling

For sample preparation, first the contents of the two retail 500 g VP samples taken from each fresh whey cheese batch were combined aseptically into a pre-sterilized glass container after removal of their films under a laminar flow hood. This was carried out to account for potential existence of variability in the post-process microbial contamination levels within retail VP of each batch, as well as for the practical reason to obtain in excess (1000 g) the fresh whey cheese quantity required for each trial (batches A to D). Then, 50 g portions of soft cheese mass of each batch were transferred by weighing, with the aid of pre-sterilized stainless-steel spatulas, into new clean vacuum bags of small size suitable for food storage (Cryovac BK3550 bag; Food Care, Sealed Air Corporation, Milano, Italy). Half of the bags were vacuumsealed directly (vacuum: minus 1 bar; 99.9%) using a MiniPack-Torre, model MVS45L vacuum sealing machine (Dalmine BG, Italy) to serve as fresh whey cheese control (CN) samples, whereas the remaining half bags were VP as above, following addition of 5% CEntE, i.e., 2.5 mL in each bag with 50 g cheese, and massaged by hand for 30 s from outside the bag to evenly distribute the active enterocins before sealing. The CEntE was previously thawed at room temperature. Portions of the same CEntE were used in all cheese trials to exclude variations in enterocin activity. The rest of each portion was well-assay tested against *L. monocytogenes* no.10 [35,36] to ensure no reduction in the CEntE titer, which was 400 AU/mL and remained at this level after storage at −30 °C for at least nine months (data not shown). All VP samples were stored in a refrigerated incubator (VelpScientifica FOC 225I, Usmate, Milano, Italy) at 4.0 ± 0.1 °C and analyzed microbiologically and for pH at 0, 8, 15, 30 and 40 days of storage. A 40-day storage period at 4 °C was consistent with the retail shelf life of Anthotyros cheese products labeled on their original VP bags, which was 40 and 35 days for the batches A and B (Pappas plant) and batches C and D (second plant), respectively.

### 2.4. Cheese Analyses

All fresh whey cheese samples without (CN) or with CEntE were evaluated during storage for changes in pH and the evolution of natural spoilage microbiota. On each sampling occasion, each VP sample was inspected for macroscopic defects (e.g., blowing, in-package purge accumulation, spot-color changes of the cheese surface, etc.), and then it was opened aseptically near a Bunsen burner. The immediate release of unpleasant off-odors, if any, was recorded at opening. The pH of each sample was measured with a digital pH meter (Jenway 3510, Dunmow, Essex, UK). The electrode was immersed in the soft cheese mass after the microbiological sampling carried out as described below.

For microbial quantification, 10 g of whey cheese was homogenized with 90 mL of sterilized quarter-strength Ringer solution in stomacher bags (Lab Blender, Seward, London, UK) for 60 s at room temperature. The homogenates were decimally diluted with Ringer and duplicate 0.1 mL samples of the appropriate dilutions were spread on five enumeration agar media, all purchased from Neogen Culture Media (formerly Lab M, Heywood, Bury, UK). Specifically, the populations of total mesophilic cheese spoilage microbiota were enumerated on milk plate count agar (MPCA) incubated at 37 °C for 48–72 h, whereas the populations of total psychrotrophic spoilage microbiota were enumerated on tryptone soya agar with 0.6% yeast extract (TSAYE) incubated at 12 °C for 5–7 days. Selective enumerations of the Gram-negative bacteria counts were performed on the high dilution MPCA/37 °C and TSAYE/12 °C plates for most cheese samples. This was necessary, and also quite easy to carry out, because the large and glistering Gram-negative bacteria colonies could be distinguished macroscopically from the small and creamy LAB-like colonies on MPCA and TSAYE plates, as previously illustrated by Samelis and Kakouri [38]. The populations of total cheese spoilage LAB were also enumerated comparatively on MRS agar incubated at 30 °C for 72 h, while the populations of pseudomonads and other related Gram-negative spoilage bacteria were selectively enumerated on *Pseudomonas* agar with cetrimide-fucidin-cephaloridine (CFC), incubated at 25 °C for 48 h. Prevalent *Enterobacteriaceae* grew well on MPCA/37 °C and/or TSAYE/12 °C media, which facilitated their selective colony enumeration and isolation for characterization. Enterococci, staphylococci and yeasts were not analyzed in the course of this study because preliminary shelf-life Anthotyros trials showed that the above microorganisms were at low (<5log) levels throughout VP storage at 4 °C (unpublished data).

### 2.5. Isolation of Representative Colonies of the Dominant Whey Cheese Spoilage Microbiota

The terminal spoilage microbiota of VP whey cheese samples after 40 days of VP storage at 4 °C was abundant on MPCA/37 °C, MRS/30 °C and TSAYE/12 °C and consisted mainly of presumptive LAB colonies, which were always prevalent (see Results). Accordingly, five random LAB-like (i.e., whitish, creamy, small to pinpoint) colonies were picked from one highest-dilution plate of the above three media for each CN or CEntE sample on day 40. This constant LAB isolation protocol resulted in the collection of 120 presumptive LAB colonies in total (30 from each whey cheese batch A to D; 40 from each agar medium), which were transferred for growth in 10mL of MRS broth (pH 6.4 ± 0.2) and incubated at 30 °C for 72 h, unless growth of the isolate was earlier.

In parallel, one to five presumptive colonies (i.e., large, glistering, white, grayish or yellowish) of Gram-negative spoilage bacteria were randomly picked from MPCA/37 °C, TSAYE/12 °C and respective CFC/25 °C agar plates of most whey cheese samples on day 30 and 40 of storage. Contrary to LAB, the isolation protocol of Gram-negative colonies could not be constant because of major fluctuations in their populations on high dilution MPCA and TSAYE plates from day 30 to 40. Eventually, 96 colonies were collected: 62, 14 and 20 were isolated from CFC, MPCA and TSAYE plates, respectively. No Gram-negative colonies were recovered from MRS agar. All presumptive Gram-negative bacteria isolates were cultured in 10mL brain heart infusion (BHI) broth (pH 7.4 ± 0.2) at 25 °C for 72 h, unless growth was earlier. Then, all bacterial isolates were checked for purity by streaking on MRS agar (pH 5.7 ± 0.1) or BHI agar (pH 7.4 ± 0.2) and incubated at 30 °C for 72 h or 25 °C for 48 h, respectively. Any LAB-like, acetate-sensitive MPCA or TSAYE isolates growing poorly on MRS agar were cultured in the BHI media onwards. The purified isolates were stored in MRS or BHI broth with 20% (*w*/*v*) glycerol at −30 °C.

### 2.6. Biochemical Characterization of Whey Cheese Spoilage LAB Isolates

Presumptive LAB (MRS, MPCA or TSAYE) colony isolates were first confirmed for Gram-positive and catalase-negative reactions by the rapid 3% KOH and 3% hydrogen peroxide testing methods, respectively, and then were grouped according to basic biochemical taxonomic criteria at the genus or species level, as most recently described by Samelis et al. [39]. Unless stated otherwise, the incubation temperature was 25 °C for all biochemical tests. Grouping of the LAB isolates was based on cell morphology by phase contrast microscopy, growth at 37 °C and 45 °C in MRS or BHI broth, gas (CO_2_) production from glucose, ammonia (NH_3_) production from arginine, growth in MRS broth with 4% and 6.5% NaCl, slime formation from sucrose and fermentation of 13 key differentiating sugars (Merck or Serva) in pre-sterilized 96-well miniplates, according to Samelis et al. [39]. The entire sugar fermentation profiles of representative LAB isolates were determined by the API 50 CHL identification kit (BioMerieux, Marcy l’ Etoile, Lyon, France), according to the manufacturer instructions.

### 2.7. Molecular Identification of Representative Whey Cheese Spoilage LAB Isolates

The identities of a total of 17 representative isolates of the most prevalent whey cheese LAB species, as well as their biotypes based on the biochemical characterization results, were confirmed by 16S rRNA sequencing analysis. The selected isolates were subcultured overnight in MRS broth (25 °C), and 1.5 mL from the culture was used for a modified DNA extraction method, according to Tsafrakidou et al. [40]. The pellet containing the DNA of each LAB isolate was suspended in 100 μL TEbuffer (pH 8.0, 50 mM Tris-HCl, 20 mM EDTA) and kept at −20 °C until the analysis.

The V1–V9 region of the 16S rRNA gene (ca. 1500 bp) was amplified using the universal bacterial primers 8F (5′-AGAGTTTGATCCTGGCTCAG-3′) and 1492R (5′-CGGTTACCTTGTTACGACTT-3′). Amplification reactions were prepared using theKapa Taq PCR kit (Kapa Biosystems Inc., Worburn, MA, USA) according to the manufacturer instructions using 50 ng of template DNA and a reaction volume of 50 μL. PCR was performed in the DNA Engine Peltier Thermal Cycler (BioRad) using the following conditions: initial denaturation at 95 °C for 3 min, 35 cycles of denaturation at 95 °C for 30 s, annealing at 58 °C for 30 s, extension at 72 °C for 1 min followed by a final extension at 72 °C for 2 min. PCR products were separated in 1.2% agarose gel stained with ethidium bromide and purified using the PCR clean-up Gel extraction kit (Macherey-Nagel, Düren, Germany). Sanger sequencing of the amplified products was performed using the primers 8F (5′-AGAGTTTGATCCTGGCTCAG-3′), 515FB (5′-GTGYCAGCMGCCGCGGTAA-3′) and 1492R (5′-CGGTTACCTTGTTACGACTT-3′) by CeMIA (Larissa, Greece). Sequencing trace files were analyzed and assembled into consensus sequences using the GEAR genome analysis server (gear-genomics.com) (accessed on 15 September 2021). Taxonomic analysis was performed by using the GenBank BLAST program at the NCBI website.

### 2.8. Biochemical Identification of Whey Cheese Spoilage Gram-Negative Isolates

The Gram-negative isolates were also tested for Gram reaction, as above, and for oxidase production by smearing cells of each colony on filter paper wetted with a few drops of a 1% tetramethyl-p-phenylenediamine solution [40]. Only the Gram-negative colonies, which were either oxidase-positive (i.e., the filter paper was purpled in seconds) or oxidase-negative, were tentatively identified at the genus or species by using the API 20E kit and the corresponding biochemical identification code manual (BioMerieux), according to the manufacturer’s instructions. No molecular identifications of Gram-negative spoilage bacterial isolates were performed in the course of this study.

### 2.9. Statistical Analysis

As mentioned, four independent batches (A to D) of fresh Anthotyros whey cheese products, considered as individual replicates (*n* = 4), were analyzed for two process treatments, without (CN) or with enterocin addition in the form of CEntE. The microbiological data were converted to log CFU/g and, along with the data for pH, were subjected to a one-way analysis of variance using the software Statgraphics Plus for Windows v. 5.2 (Manugistics, Inc, Rockville, MD, USA). The means were separated by the least significant difference procedure at the 95% confidence level (*p* < 0.05) for determining the significance of differences in each treatment, with time of storage and between the two treatments (CN vs. CEntE) on each sampling interval.

## 3. Results and Discussion

### 3.1. Evolution of the Whey Cheese Spoilage Microbiota in Relation with the Cheese pH Changes during Storage

The results of the microbial quantification analyses along with the pH changes during storage of the four Anthotyros whey cheese batches in the absence (CN) or presence of the CEntE are summarized in Table 1 and are also tabulated separately for each batch in the Tables S1–S5. Evidently, the initial high mean pH 6.8 of all fresh VP whey cheese batches on day zero underwent major progressive reductions during storage at 4 °C for 40 days, apparently due to an unmonitored natural acidification caused by adventitious LAB and, potentially, additional fermentative bacterial contaminants that were able to grow at refrigeration temperatures. Specifically, the pH was practically unchanged during the first 8 days of storage (*p* > 0.05). However, it was reduced (*p* < 0.05) to mean values of pH 6.0–6.2 on day 15, while further major decreases (*p* < 0.05) to mean pH values of 4.6–5.1 were measured in all cheese samples by the end of storage (Table 1). The strongest (pH 4.44) and the mildest (pH 5.42) natural acidification of the whey cheeses on day 40 were measured in batch D with CEntE and in batch B without enterocin (CN), respectively (Table S1). Similar batch-dependent fluctuations in the pH reduction pattern occurred from day 15 onwards between batches A to D, as well as between the CN and CEntE samples of each batch overall. The addition of the CEntE significantly (*p* < 0.05) enhanced the natural acidification of the VP whey cheese products after 30 to 40 days of storage under refrigeration (Table 1).

**Figure 1:**
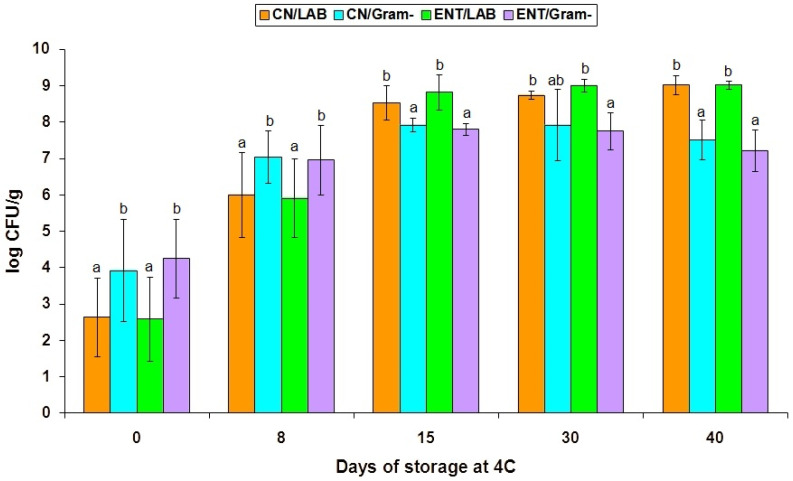
Comparative growth of LAB and Gram-negative spoilage bacteria during refrigerated storage of fresh VP Anthotyros whey cheeses without (CN) or with crude enterocin A-B-P extract (ENT), based on selective enumerations of their colony counts grown on TSAYE plates incubated at 12 °C for 7days. Whiskers for each bar indicate standard deviations. Within each storage day, whiskers lacking a common lowercase letter (a, b) are significantly different (*p* < 0.05).

Microbiologically, major growth increases (*p* < 0.05) of the initial (day zero) populations of the total mesophilic (MPCA/37 °C), psychrotrophic (TSAYE/12 °C), LAB (MRS/30 °C) and Gram-negative (CFC/25 °C) whey cheese spoilage microbiota occurred after only one week of VP storage at 4 °C. Despite the fact that the pH was unchanged from day zero to eight, the spoilage bacteria that were enumerated on the above agar media grew abundantly by an average of 1.4 to 3.35 log CFU/g, irrespective of the absence or presence of the CEntE extract in the whey cheese mass (Table 1). Hence, on day eight, the mean populations of total LAB on MRS (2.91–2.93 log increase) and Gram-negative *Pseudomonas*-like bacteria on CFC (2.88–3.02 log increase) exceeded five and six log units, respectively. Correspondingly, the total psychrotrophic bacteria on TSAYE at 12 °C (2.95–3.35 log increase) exceeded the 7-log food spoilage threshold, whereas the lowest mean increases of 1.4–2.53 logs were determined for total mesophilic bacteria on MPCA at 37 °C (Table 1). These results indicated that the fresh Anthotyros whey cheeses supported a rapid, abundant growth of primarily psychrotrophic spoilage bacterial contaminants, either LAB or Gram-negative bacteria, within only 8 days of VP storage at 4 °C (Table S3). Growth of all bacterial groups continued to be significant (*p* < 0.05) till day 15. However, from day 15 onwards, growth of *Pseudomonas* and related Gram-negative spoilage bacteria on the CFC agar ceased, evidently in response to the decreasing pH. With few sample exceptions (Table S5), CFC populations remained below 8 log CFU/g and eventually declined, particularly in the Ent-treated whey cheese samples (*p* < 0.05) by the end the of 4 °C storage (Table 1). Conversely, LAB progressively prevailed at population levels well above 8 to 9 log CFU/g in all whey cheese batches from day 15 (batch D) to 30 and 40 (batches A, B and C) of storage (Table S4). The prevalence of spoilage LAB was most apparent on the MRS/30 °C agar (Table 1) and was confirmed by the selective enumerations of the LAB colonies correspondingly to the Gram-negative bacteria colonies on high dilution TSAYE/12 °C (Figure 1) and MPCA/37 °C (data not shown) plates. LAB populations were significantly (*p* < 0.05) higher than those of subdominant Gram-negative spoilage bacteria in all whey cheese samples on day 40 and in most of the whey cheese samples on days 15 and 30, irrespective of enterocin treatment (Figure 1 and Tables S2–S5). Although the addition of 5% CEntE extract appeared to somehow enhance LAB growth in the Ent-treated whey cheese samples compared to their untreated (CN) counterparts throughout storage, this enhancement was significant (*p* < 0.05) for the day-30 LAB populations on the MRS agar only (Table 1). Notably, a higher natural contamination and prevalent growth of Gram-negative bacteria over LAB on the TSAYE/12 °C plates during early storage (i.e., from day zero to eight; *p* < 0.05) were reversed in favor of LAB, whose populations became higher (*p* < 0.05) than those of Gram-negative bacteria on day 15 (Figure 1). A similar reversal trend in the growth pattern of main spoilage bacterial groups was also apparent, but not significant, on the MPCA plates at 37 °C (data not shown). Thus, Gram-negative, mainly psychrotrophic, bacteria promoted faster and greater growth than LAB in most fresh whey cheese samples during the first week of VP storage at 4 °C (Table 1). Accordingly, the determination of Gram-negative bacterial growth was enhanced at the low (12 °C) incubation temperature of the TSAYE plates (Figure 1).

Following the pH decreases in the naturally acidified whey cheeses after day 15 of refrigerated storage, the declines of Gram-negative bacteria were greater in batch D, followed by batches A and B produced in the Pappas Bros. plant. In contrast, batch C supported the highest survival of Gram-negative bacteria populations on the MPCA/37 °C, TSAYE/12 °C and CFC/25 °C plates during late storage, which ranged from 7.7 to 8.1 log CFU/g on day 40 (Tables S2–S5). Hence, although batches C and D were fresh whey cheese products of the same dairy plant, they displayed major variation in the growth pattern of the Gram-negative spoilage biota during storage; batches A and B varied to a lesser extent. On day 40, regarded as the sell-by-day, none of the whey cheese samples were offensively spoiled to be considered unfit for human consumption. None of the VP samples showed excessive gas blowing, accumulated viscous purge or slime or released putrid or sulfur off-odors upon opening. However, the VP film of most samples became loose after storage day 30 to 40, while most day-40 samples released a sharp acid smell, and particularly those of batch C were characterized by unpleasant malt-fermentative off-odors after day 15.

The pH and the microbial quantification results and the sensory observations of this study are in general agreement with previous relevant data on the shelf life of the Italian Ricotta [4,5,7], the Portuguese Requeijao [6,26], the Turkish Lor [24,25] and the Greek Myzithra [21], Manouri [19] and specifically Anthotyros [3,18,22,23]. Generally, spoilage of fresh whey cheeses stored in air occurs within 7–10 days, whereas VP and MAP extend the shelf life by 10–20 days at 4 °C, depending on the whey cheese properties [5,21,24]. In most of the studies above, MAP alone, or combined with abiotic preservatives such as essential oils, chitosan, Microgard and nisin [11,12,23], has been reported to be more effective than VP in delaying microbial growth and thus extending the shelf life of fresh whey cheeses. MAP reduces the growth rate of bacteria, including LAB, and inhibits the growth of *Pseudomona s* and related aerobic spoilage bacteria more than VP. On the other hand, despite the higher growth potential of Gram-negative bacteria in a vacuum, a rapid growth of LAB with a mild spoilage potential may benefit fresh whey cheese preservation, given that MAP is more costly and technologically difficult to apply in small traditional dairies. Overall, the need for the optimization of packaging characteristics, in terms of using vacuum or CO_2_ followed by refrigeration to extend the shelf-life of whey cheeses by controlling the growth of spoilage and pathogenic bacteria, has been stressed since the first relevant review by Pintado et al. [1] two decades ago. For example, VP/4 °C storage was very effective in inhibiting growth of aerobic yeasts, staphylococci and, surprisingly, anaerobic spore-forming clostridia in Requeijão [6]. Positive effects were attributed to relevant acidification of the VP Requeijão samples caused by the adventitious lactose-fermenting LAB, which were not identified in those studies [1,6]. As mentioned, we also observed growth inhibitory effects against aerobic yeasts and catalase-positive cocci or bacilli (i.e., occasionally detected at levels 2–4 log cfu/g on day zero) during VP/4 °C storage. Specifically, typical irregular colonies of catalase-positive sporoforming bacilli were ca. 3.5 log CFU/g in the fresh (day zero) samples of batch D, but those ‘stinky’ colony contaminants disappeared from all countable MPCA and TSAYE plates on day eight (unpublished data). Natural *Bacillus*, *Paenibacillus* and *Clostridium* contaminants also occurred in industrial Ricotta and Ricotta salad, and a *Bacillus mycoides* strain contributed to industrial cheese spoilage by causing a pink discoloration [7,41].

Contrary to staphylococci and catalase-positive bacilli, neither the growth of *Pseudomonas* and closely related bacteria on CFC (Table 1) nor that of the total Gram-negative bacteria (Figure 1) was suppressed in Anthotyros before day 15 of VP storage, confirming the results of most fresh whey cheese studies cited above. For instance, Angelidis et al. [42] reported mean non-LAB levels as high as 6.55 log units in market Greek whey cheese products. Kalogridou et al. [18] found mean 6.74 log counts of coliform bacteria in Anthotyros, while Manouri contained mean levels of total *Enterobacteriaceae* as high as 5.37–5.89 and 7.26–8.32 log CFU/g in the interior and the surface of market cheeses, respectively, after 20 days of storage at 4 °C [19]. Recently, Pappa et al. [43] found mean total *Enterobacteriaceae* countsas high as 6.00 and 5.82 log CFU/g in sheep and goat Urda, a traditional whey cheese made in Northern Greece and other Balkan countries, after 25 days of ripening at 19 °C. The high growth potential of the natural *Enterobacteriaceae* contaminants, which, respectively, were 4.64 and 3.73 log CFU/g in the fresh (day one) sheep and goat Urda cheese samples, was highlighted [43].

Regarding the CEntE effects, the final populations of Gram-negative bacteria enumerated on CFC declined significantly more in the Ent-treated than the CN whey cheeses (Table 1), probably because the greater pH reduction in the former cheeses enhanced in situ activity of the enterocin A-B-P molecules [36], in addition to stimulating LAB growth and possibly selecting for different LAB types than in the CN samples. Although several enterocins have been reviewed to inhibit Gram-negative bacteria in vitro [28,33], previous studies on the use of enterocins to control fresh whey cheese spoilage are scarce, if any. Most direct enterocin applications refer to fresh acid-curd cheeses or other fermented cheese products made of raw or pasteurized milk, where the fast pH drop by the high (>7–9 log units) starter LAB growth within the first 24h of cheese manufacture creates quite a stressful cheese environment against all non-LAB contaminants [28,30,31,34]. Conversely, bioprotective mixed LAB cultures with *Enterococcus* have been applied to control whey cheese spoilage [14], but their performance will be discussed following presentation of the LAB and Gram-negative bacterial characterization data below.

### 3.2. Biochemical Characterization and Distribution of the LAB Biota in Anthotyros Cheeses

In total, 96 out of the 120 LAB colonies isolated from the whey cheese samples on day 40 were obligatory heterofermentative (gas-forming), arginine-negative coccobacilli, able to grow at 37 °C, but not at 45 °C, and in 6.5% salt (Table 2). Thus, they were typical *Leuconostoc*-like bacteria [44,45] and represented the terminal spoilage LAB group of the traditional Anthotyros cheese products of this study. Indeed, *Leuconostoc* spp. accounted for 80% of the total LAB isolates, were evenly isolated from the CN or CEntE samples of all whey cheese batches A to D (Table 2) and were psychrotrophic because they grew predominantly at 4 °C at final populations above 8 to 9 log CFU/g in all VP whey cheeses (Table 1). Interestingly, most of them (79 isolates; 65.8%) failed to produce slime (dextran) from glucose in vitro, a typical property of several (dairy) *Leuconostoc* spp. [44]. Moreover, most (15 out of 17) of the subdominant (14.2%) slime-producing *Leuconostoc* isolates were recovered from batches A and B of the Pappas dairy, mainly from batch A (Table 2). Notably, the addition of 5% CEntE did not have any major effects on altering the numerical distribution of the predominant non-slime-producing *Leuconostoc* spp. isolates or of the subdominant slime-producing *Leuconostoc* spp. and the other types of LAB isolates, compared to the CN samples of all whey cheese batches (Table 2). Therefore, the numerical distribution of the LAB isolates at the species level will be presented and discussed for each cheese, batch A to D only, in all following biochemical characterization tables.

The 96 *Leuconostoc* isolates were split further into five distinct biotypes based on their fermentation reactions with six key sugars and ability to form slime (Table 3). Importantly, all 96 isolates fermented lactose and galactose strongly, consistent with their dairy (whey cheese) habitat, as well as D-xylose and trehalose with the exception of the four isolates in biotype L5 (Table 3). Biotype L5 also failed to ferment L-arabinose.However, it fermented raffinose. Overall, biotype L5, which was subdominant of the other biotypes L1–L4, isolated from batches C and D only, was clearly different from them at the species level; it gave perfect biochemical identification as *Leuconostoc lactis* [45].

The remaining 92 xylose-positive and trehalose-positive *Leuconostoc* isolates in biotypes L1–L4 gave variable fermentation reactions with L-arabinose and raffinose (Table 3). The first two most prevalent biotypes, L1 and L2, gave negative and positive reactionswith both sugars, respectively, while biotype L3 fermented L-arabinose but not raffinose. Moreover, all 75 isolates in biotypes L1, L2 and L3 did not form slime, contrary to biotype L4,which included all slime-positive *Leuconostoc* isolates; the majority of them (15 out of 17) fermented L-arabinose and raffinose strongly. Overall, based on their phenotypic properties in Table 3, the slime-positive biotype L4 was biochemically identified as *Leuconostoc mesenteroides*, the L-arabinose-positive but slime-negative biotypes L2 and L3 might be *Leuconostocpseudo mesenteroides* or atypical *Leuconostoc mesenteroides* [44,45], whereas the most numerous L-arabinose-negative biotype L1 lied phenotypically within three closely related species commonly isolated from dairy foods, *L. mesenteroides*, *L. pseudomesenteroides* and *Leuconostoc argentinum*, originally detected in raw milk [46]. *L. argentinum* resembles phenotypically with members of the *L. mesenteroides* group that are unable to produce slime (Table 3), while genotypically it has been reclassified as a later synonym of *L. lactis* [47]. On the other hand, *L. mesenteroides* is taxonomically a very complex species that currently includes four subspecies, *mesenteroides*, *dextranicum*, *cremoris* and *jonggajibkimchi*, while its former fifth subspecies, *L. mesenteroides* subsp. *suionicum* [48], was recently raised to the species level by Jeon et al. [49]. The subspecies of *L. mesenteroides* are highly intermixed phenotypically, and thus it is impossible to differentiate them by biochemical criteria, except of the slime-negative *L. mesenteroides* subsp. *cremoris* [44,45,48,49]. However, solely on their phenotype basis, none of the biotypes L1 to L4 were perfectly identifiable as *L. mesenteroides* subsp. *cremoris* because this dairy starter subspecies is oligo-fermenting, typically failing to ferment L-arabinose, D-xylose and trehalose (Table 3), plus sucrose, which was fermented by all *Leuconostoc* isolates of this study (data not tabulated). Thus, representatives of the biotypes L1 to L5 (Table 3) required molecular analyses to assure their accurate identification at the species level.

Typical *Carnobacterium* isolates (i.e., single slender rods forming little or no gas from glucose and ammonia from arginine normally or weakly, but being unable to form slime from sucrose and grow at 45 °C or in 6.5% salt) [50] were the main subdominant spoilage LAB group with a total isolation frequency of 10.9%, underneath the predominant (80%) *Leuconostoc* isolates (Table 2). However, all *Carnobacterium* (in total 13 isolates) were recovered from batches B and C only. Hence, the ability of psychrotrophic, non-aciduric carnobacteria to grow competitively and eventually survive at high numbers in the low pH whey cheese products at the end (day40) of VP storage at 4 °C was batch-dependent rather than plant-dependent. Because the natural occurrence of *Carnobacterium*, one of the most typical meat-specific spoilage and/or protective LAB [51], in dairy foods is of special biotechnological interest, all isolates were biochemically identified with the API50 CHL method and proved quite diverse, since they were split into four biotypes (C1 to C4) according to the corresponding sugar fermentation reactions reported in the *Bergey’s Manual* for the differentiation of *Carnobacterium* sp. (Table 4).

All seemed to be phenotypic intermediates of the species *Carnobacterium gallinarum*, which ferments lactose, melezitose, tagatose and D-xylose but not inulin, mannitol and melibiose, and *Carnobacterium maltaromaticum*, which typically ferments the above sugarsvice versa [50]. However, *C. maltaromaticum* is the only species of the genus so far associated with milk and milk products [50]. This fact is consistent with the ability of all *Carnobacterium* whey cheese isolates of this study to ferment lactose, while all failed to produce acid from inulin (Table 4), a result in contradiction with the original description of the species *C. maltraromaticum* [50]. Thus, representatives of the *Carnobacterium* biotypes C1 to C4 also required molecular analyses to assure their identification at the species level.

The remaining 11 LAB isolates were sporadic and seemed unrelated with the terminal aciduric spoilage of VP whey cheese samples at 4 °C. Seven of them were thermophilic *Streptococcus* isolates (Table 2) that fermented lactose and sucrose only and thus were typical *S. thermophilus* (data not tabulated) [39]. All were recovered from the MPCA/37 °C plates of the whey cheese batch D (Table 2) and probably were starter *S. thermophilus* strain/s that accidentally contaminated batch D at high numbers before VP and remained dormant in the cheese mass until day 40. Moreover, a single *Enterococcus* sp. was isolated from an Ent-treated sample of batch D (Table 2). It fermented L-arabinose and generally it possessed a sugar fermentation profile typical of *E. faecium* [35,39]. However, it did not display enterocin activity in the agar overlay and well assays against *L. monocytogenes* no.10 (data not shown), which meant it was a random autochthonous enterococcal strain rather than a survivor of one of the Ent+ or m-Ent+ *E. faecium* KE64, KE67, KE82 or KE118 strains used to prepare the filter-sterilized crude enterocin A-B-P extract. The remaining three isolates in Table 2 were sporadic mesophilic, obligatory homofermentative, ribose-negative LAB. Two of them were cocci sharing a sugar fermentation profile typical of certain commercial xylose-negative *Lactococcus lactis* subsp. *lactis* starter strains (data not shown), while the other isolate was an atypical *Lactobacillus* sp. requiring molecular ID tests to be identified as the species. No additional biochemical or molecular ID tests were performed for the sporadic LAB isolates during this study.

### 3.3. Identification of Representative Whey Cheese Spoilage Biotypes of Leuconostoc and Carnobacterium at the Species Level by 16S rRNA Sequencing

Thirteen and four isolates, representing all *Leuconostoc* and *Carnobacterium* biotypes in Table 3 and Table 4, respectively, were identified by 16S rRNA sequencing. Starting from the carnobacteria, the four selected isolates were WM102M (biotype C1), WM126 (C2), WM130 (C3) and WM102 (C4). All were identified as *C. maltaromaticum*; none were identified as *C. gallinarum*. Despite their phenotypic diversity (Table 4), the isolates WM126 (C2), WM102 (C4) and WM102M (C1) shared a 99.93%, 99.93% and 99.86% 16S rRNA sequence homology, respectively, with the same reference strain in BLAST, *C. maltromaticum* accession no. MH119758.1, while the isolate WM130 (C3) shared a 99.86% homology with *C. maltromaticum* accession no. KR055032.1.

With regard to the *Leuconostoc* isolates, the 16S rRNA sequencing results confirmed the identity of the representative isolate WM118 of biotype L5 as *L. lactis* (100% homology with *L. lactis* strain, accession no. MF354765.1) (Table 3). All other *Leuconostoc* isolates, 12 in total representing the biotypes L1–L4, were identified with a 100% homology as *L. mesenteroides*, despite their high phenotypic diversity and, particularly, the inability of all isolates of biotypes L1–L3 to produce slime, and, further, those of biotype L1 to ferment L-arabinose (Table 3). None of those *Leuconostoc* isolates were identified as *L. pseudomesenteroides* or *L. argentinum* by the 16S rRNA sequencing method, which further was unsuitable to distinguish the 12 *L. mesenteroides* isolates at the subspecies level. Moreover, the isolates of different biotypes were intermixed with regard to the accession no. of their closest reference strain of *L. mesenteroides* in the BLAST database: it was strain MT545072.1 for the isolates WM106, WM109B, WM136, WM137 and WM153 (L1), WM105 and WM122A (L2), WM108 (L4), MT545101.1 for the isolate WM123 (L1), and MT545113.1 for the isolates WM110A (L2), WM107 (L4) and WM121 (L3), respectively.

The vast majority of technologically important *Leuconostoc* spp. isolates from naturally or industrially fermented cheeses and other dairy products belong to the species *L. mesenteroides* [44]. However, a high phenotypic and genotypic diversity exists within dairy-associated wild *L. mesenteroides* strains that makes it difficult to discriminate them at the subspecies level [44,45,52,53]. Actually, the 16S rRNA sequencing analysis is insufficient for the differentiation of *L. mesenteroides* subsp. *Mesenteroides* and *L. mesenteroides* subsp. *Dextranicum* [45]. Analysis of additional genes, such as the *rpoB* polymorphism, and additional PCR-based (RAPD) approaches are thus required to identify *L. mesenteroides* at the subspecies level and/or to discriminate its isolates from those of *L. pseudomesenteroides* [54,55] also commonly found in dairy foods and particularly in fresh cheeses [44,56]. Moreover, recent genomic data suggest that the abovementioned 16S rRNA taxonomic insufficiency is extended to wild atypical cheese strains of *L. mesenteroides* subsp. *cremoris* that share important phenotypic traits with the other dairy subspecies of *L. mesenteroides* and *L. pseudomesenteroides*, such as the ability to produce acid from all or some of the sugars L-arabinose, ribose, D-xylose, mannitol, cellobiose, maltose, melibiose, sucrose, trehalose, raffinose and turanose [56]. These findings are contradictory with the effective taxonomic description(s) of the dairy starter reference strain(s) of *L. mesenteroides* subsp. *cremoris*. Interestingly, the prevalent, non-slime-forming whey cheese isolates in biotypes L1-L3 of *L. mesenteroides* (Table 3) were similar phenotypically with the fresh raw milk cheese isolates of *L. mesenteroides* subsp. *cremoris* described by Pogacic et al. [56]. All had strong capability for lactose, galactose, glucose and D-xylose fermentation and generally appeared to be highly adapted for growth in the whey cheese niche and at refrigeration temperatures. Particularly, the predominant biotype L1 isolates were oligo-fermenting compared to typical *L. mesenteroides* isolates from foods. The application of more advanced molecular taxonomic methods for the exact subspecies allocation of the *L. mesenteroides* whey cheese isolates was beyond the scope of this study.

The primary micro-ecological and biotechnological finding of this study was that diverse strains of *L. mesenteroides* accounted for 76.7% of the terminal spoilage LAB biota in fresh VP Anthotyros whey cheeses stored at 4 °C, followed by *C. maltaromaticum* (10.9%) (Table 5). The LAB association was batch-dependent and plant-dependent, with the Anthotyros (batches A and B) product of Pappas Bros. harbouring mostly *L. mesenteroides* and generally being less diversified than the Anthotyros (batches C and D) product of the second dairy (Table 5). Additionally, the MRS agar was more selective for the isolation of *L. mesenteroides*, whereas the considerably fewer *C. maltaromaticum* and *L. lactis* isolates were recovered from TSAYE and MPCA only (Table 5). In contrast, as it was stressed above, supplementation of the fresh whey cheese with 5% CEntE did not alter the spoilage LAB species distribution in any of the four batches tested (viz. Table 2).

### 3.4. Biochemical Identification of Gram-Negative Spoilage Bacterial Isolates

The 96 Gram-negative whey cheese spoilage isolates recovered from CFC, MPCA and TSAYE plates were characterized at the genus or species by the API20E identification kit. Most of them (74 isolates) were oxidase-negative, fermentative members of the genera *Hafnia*, *Serratia*, *Rahnella*, *Enterobacter*, *Klebsiella* and *Pantoea* (Table 6). Additional 20 isolates were oxidase-positive, non-fermentative members of the genera *Pseudomonas* and *Aeromonas*, all recovered from CFC (Table 7). The remaining two were non-fermentative, although oxidase-negative isolates; their only positive reaction was citrate utilization, resulting in an acceptable identification as *Flavibacterium oryzihabitans* (Table 7). Because the API20E method has been developed for the identification of oxidase-negative, fermentative members of the *Enterobacteriaceae* family, additional biochemical tests and molecular taxonomic tools are required to accurately identify the present *Aeromonas* and *Pseudomonas* isolates at the species level (Table 7). Conversely, very good to excellent species identification was recorded for the two primary Gram-negative whey cheese spoilage bacteria on days 30 and 40 of VP storage at 4 °C, *Hafnia alvei* and *Serratia liquefaciens*, which together comprised 60.4% (58 out of 96) of the total Gram-negative isolates (Table 8). Specifically, two distinct biotypes of *H. alvei*, biotype I (Table 6) exclusively isolated as the predominant spoiler from batch C (Table 8) and biotype II (Table 6) exclusively isolated from batch D (Table 8), were detected. Similarly, two distinct biotypes of *S. liquefaciens*, biotype I (Table 6) exclusively isolated from batch C (Table 8) and biotype II (Table 6) exclusively isolated from the Pappas Bros. whey cheese batches A and B (Table 8), were detected. Moreover, all *Rahnellaaquatilis* isolates were recovered from batches A and B (Table 8), particularly from the CFC plates on day 30, and were succeeded by *S. liquefaciens* biotype II and *Pseudomonas* in the same batches on day 40 (data not shown). Conversely, all *Aeromonas* isolates were recovered from the CFC agar plates of batch D, whereas few *Klebsiella oxytoca, Enterobacter* sp./*E. cloacae* and *Pantoea* sp. isolates were detected in batches C and D, but not in batches A and B produced in the Pappas Bros. plant (Table 8). Altogether, the above findings suggest a strong plant-dependent rather than batch-dependent persistence of specific spoilage Gram-negative bacteria types in fresh Anthotyros whey cheeses. In this regard, the most prominent difference was the total absence of *Hafnia* isolates from batches A and B, as opposed by the total absence of *Pseudomonas* isolates from batches C and D, respectively (Table 8). Conversely, the supplementation of the four fresh whey cheese batches with 5% CEntE did not have any major effects on altering the species distribution of Gram-negative spoilage isolates in the Ent-treated samples compared to the respective CN samples (data not tabulated). Therefore, the distribution of Gram-negative bacteria is presented as the total number of isolates of each species from each whey cheese batch, A to D, in Table 8.

Biotechnologically, the most important difference between *S. liquefaciens* biotype II (batches A and B) and biotype I (batch C) was the ability of the former to hydrolyse gelatine (Table 6), a typical characteristic reaction of this species. The *H. alvei* biotypes I and II differed only in the ability of the former to utilize citrate strongly. Apart from citrate utilization, *H. alvei* biotype I and both biotypes of *S. liquefaciens* showed a high ability to decarboxylate lysine and ornithine in vitro, properties potentially associated with spoilage pathways of these Gram-negative speciesin situin fresh whey cheeses, particularly in those of batch C herein. Moreover, all fermentative Gram-negative isolates in Table 6, except of a few *H. alvei* biotype I or II isolates, formed acetoin strongly, an additional reaction potentially involved in fresh whey cheese spoilage by enterobacteria. In contrast, none of the Gram-negative isolates produced hydrogen sulphidein vitroor possessed tryptophane deaminase activity (Table 6 and Table 7).

Members of the genera *Enterobacter* (*E. cloacae*, *E. aerogenes*) and *Hafnia*, along with *Citrobacter freundeii*, were reported to occur in Ricotta [1], while more recent studies consider *Pseudomonas* as the main Gram-negative spoiler of Ricotta fresca cheeses [5,14,29]. However, detailed molecular identification studies on the predominant *Pseudomonas* spp. in the spoilage community of fresh whey cheeses are still missing. Conversely, blue pigment-producing *Pseudomonas fluorescens* strains were identified as the main spoiler in fresh Italian mozzarella cheese, causing the ‘blue mozzarella’ defect [57,58]. In this study, all *Pseudomonas* isolates from batches A and B produced a yellowish-bluish pigmentation in vitro and were broadly characterized as *P. fluorescens/putida* by the API20E ID manual. Thus, these strains may also be usable for in situ pigment production in fresh Greek whey cheeses. Nevertheless, an early study by Tzanetakis and co-workers, cited in Pintado et al. [1], detected *Enterobacter*, *Klebsiella* and *Citrobacter* spp. in 84%, 85% and 75% of Greek Anthotyros, Myzithra and Manouri samples, respectively. In a later study [19], *Hafnia* (68.75%) dominated over the total *Enterobacteriaceae* spoilage biota of Manouri cheeses stored for 20 days at 4 °C. Overall, advanced microbiological ecology studies on the Gram-negative spoilage association of Greek whey cheeses do not exist; limited information is provided in recent reviews by Litopoulou-Tzanetaki and Tzanetakis [15,20].

In summary, although growth of *Pseudomonas/Aeromonas* and *Enterobacteriacae* could not be suppressed before day 15, the increasing growth competition and prevalence of *L. mesenteroides*, *L. lactis* and *C. maltraromaticum* exerted a mitigation effect against the Gram-negative spoilage community in the Anthotyros samples after day 15 of VP/4 °C storage. Significant growth inhibitory or mitigation effects against Gram-negative spoilage bacteria have recently been attributed to the use of *Carnobacterium* spp./*C. maltaromaticum* and/or *Lacticaseibacillus rhamnosus* in the form of single selected adjunct strains or commercial bioprotective cultures (i.e., Lyofast CNBAL, Lyofast FPR2) in fresh MAP packed Ricotta fresca cheese [14,29] and another Italian fresh cheese type [59]. Specifically, Lyofast CNBAL containing *Carnobacterium* spp. was the best-performing commercial culture in fresh Ricotta by delivering a maximum reduction of 1.93 and 2.66 log CFU/g in the populations of *Pseudomonas* and *Enterobacteriaceae*, respectively, after 14 days of MAP storage at 4 °C [14]. Similar results were obtained by CNBAL in Ricotta fresca cheeses that were surface-contaminated with *Pseudomonas* spp. and then stored at 4 °C in MAP for 21 days; the low acidification properties of *Carnobacterium* spp. caused limited variation of pH and no changes in water activity, fat and protein contents compared to the fresh MAP Ricotta cheeses without CNBAL [29]. Moreover, *C. maltromaticum* CNB06 and *L. rhamnosus* RH05 lowered psychrotrophic Gram-negative bacteria of almost 3 log CFU/g in another Italian fresh cheese after 5 weeks of MAP storage at 8 °C [59]. In contrast, neither Lyofast FPR2, including *E. faecium* and mesophilic lactobacilli, nor the fermentate MicroGARD 430 or *Latilactobacillus sakei* LSK04 were effective in controlling growth of Gram-negative bacteria in MAP Ricotta and fresh Italian cheese [14,59]. The probiotic species *Bifidobacterium animalis* fully inhibited growth of *Ps. aeruginosa* in whey cheese, whereas a probiotic *Lactobacillus casei* strain provided the least inhibition [60].

## 4. Conclusions

This is the first detailed microbiological ecology study on the evolution of specific spoilage bacterial types prevailing in traditional Greek fresh Anthotyros whey cheese stored at 4 °C in a vacuum for up to 40 days and the effects of adding a crude enterocin A-B-P-containing extract (CEntE) on the whey cheese spoilage association. Overall, psychrotrophic Gram-negative bacteria, primarily *Hafnia* spp., *Serratia* spp., *Pseudomonas* spp. and *Aeromonas* spp., promoted faster growth than LAB at the beginning and until day15 of storage. However, LAB outgrew the Gram-negative bacteria and prevailed in all whey cheeses on later storage days by causing a strong or milder batch-dependent natural acidification. Two major atypical non-slime-forming biotypes of *Leuconostoc*-like bacteria, molecularly identified as *L. mesenteroides*, dominated the terminal whey cheese spoilage association, which also included two minor typical *L. mesenteroides* biotypes plus four subdominant biotypes of *C. maltaromaticum*, one *Leuconostoc lactis* biotype and sporadic isolates of three homofermentative LAB species. The evolution and relative distribution of LAB and Gram-negative bacteria species were strongly batch- and plant-dependent. The CEntE neither retarded growth nor altered the whey cheese spoilage association, but it enhanced LAB growth and the declines of Gram-negative bacteria by late storage without affecting the Gram-negative bacteria species surviving at spoilage in each cheese batch. Hence, the possible advantages of using the CEntE in fresh whey cheese include an increased inactivation of Gram-negative spoilers in favor of LAB, along with an increased inactivation or growth inhibition of *L. monocytogenes* that has yet to be assessed. To our knowledge, this is the first report specifying atypical non-slime-producing strains of *L. mesenteroides* as the primary aciduric LAB spoilers of a fresh whey cheese and the first ever isolation of indigenous *C. maltaromaticum* strains from a commercial Greek dairy product. Additional taxonomic studies are in progress to identify the psychrotrophic *L. mesenteroides* whey cheese spoilage isolates at the subspecies level, plus biochemical characterization studies for the selection and application ofwild *L. mesenteroides*, *C. maltaromaticum* or *L. lactis* strains as a biopreservative and/or protective cultures against *L. monocytogenes* and other pathogens in fresh Greek whey cheeses.

## Figures and Tables

**Table 1:** Growth of the main spoilage bacterial groups (log CFU/g) in association with the pH changes during refrigerated (4 °C) storage of fresh, vacuum packaged Anthotyros whey cheeses with (CEntE) or without (CN) addition of 5% crude enterocin A-B-P extract ^a^.

Bacterial Group	Cheese Treatment	Days of Storage				
		0	8	15	30	40
Total mesophilic dairy bacteria	CN	4.45 a (1.20)	6.98 b (0.92)	7.79 bc (0.71)	8.48 c (0.28)	8.68 c (0.52)
	CEntE	4.89 a (1.38)	6.29 b (1.21)	8.37 c (0.60)	8.81 c (0.40)	8.66 c (0.30)
Total psychrotrophic bacteria	CN	3.92 a (1.40)	7.27 b (0.49)	8.64 cd (0.40)	8.87 d (0.15)	9.03 d (0.28)
	CEntE	4.25 a (1.08)	7.20 b (0.61)	8.93 c (0.38)	9.05 c (0.14)	9.04 c (0.12)
Total lactic acid bacteria (LAB)	CN	2.64 a (1.08)	5.57 b (1.23)	7.21 c (0.85)	8.49 cd * (0.34)	8.86 d (0.24)
	CEntE	2.58 a (1.15)	5.49 b (1.35)	7.42 c (0.86)	8.94 d * (0.19)	8.89 d (0.12)
*Pseudomonas*-like and related Gram-negative bacteria	CN	3.35 a (1.29)	6.37 b (0.76)	7.84 c (0.42)	7.41 cb (1.48)	6.68 cb * (1.37)
	CEntE	3.40 a (1.25)	6.28 b (0.75)	7.79 c (0.33)	7.21 cb (0.61)	5.51 ab * (2.05)
Whey cheese pH	CN	6.80 d (0.18)	6.84 d (0.28)	6.21 c (0.13)	5.51 b * (0.23)	5.14 a * (0.21)
	CEntE	6.83 c (0.14)	6.82 c (0.23)	5.98 b (0.54)	4.87 a * (0.28)	4.63 a * (0.21)

a Values are the means of four independent whey cheese batches (*n* = 4); standard deviation values are shown in brackets. Within a row, means lacking a common lowercase letter (a, b, c, d) are significantly different (*p* < 0.05). Within a column for each h analysis, means bearing an asterisk are significantly different (*p* < 0.05).

**Table 2:** Biochemical characterization and basic grouping of 120 spoilage LAB isolates from Anthotyros whey cheeses, their numerical distribution in each of the four cheese batches with (Ent) or without (CN) addition of 5% crude enterocin extract and their total percent isolation frequency, after storage at 4 °C in vacuum packages for 40 days.

LAB Genus/Phenotypic Group	Basic Differentiating Characteristics								Whey Cheese Batch/Treatment								Total Isolates
									A		B		C		D		(% frequency)
	MA	CO_2_	NH_3_	15 °C	37 °C	45 °C	6.5%	SL	CN	Ent	CN	Ent	CN	Ent	CN	Ent	
*Leuconostoc*-likebacteria Unable to form slime	CB	+	−	+	+	−	+/++	−	10	8	11	10	7	11	10	12	79 (65.8)
*Leuconostoc*-likebacteria Slime producers	CB	+	−	+	+	−	++	+	4	7	2	2	2				17 (14.2)
*Carnobacterium*	SR	(+)/−	+/(+)	+	V	−	−	−			2	3	4	4			13 (10.9)
Thermophilic *Streptococcus* spp.	LC	−	−	−	+	+	−	−							5	2	7 (5.9)
*Enterococcus* spp.	C	−	+	+	+	+	++	−								1	1 (0.8)
Mesophilic, homofermentative arginine-negative LAB cocci	C	−	−	+	+	−	−	−					2				2 (1.6)
Mesophilic, homofermentative arginine-negative lactobacilli	R	−	−	+	+	−	+	−	1								1 (0.8)
Total isolates from each batch									15	15	15	15	15	15	15	15	120

ΜA, microscopic appearance as rods (R), small slender rods (SR), cocci (C), large cocci (LC) or coccobacilli (CB); CO_2_, gas production from glucose; NH_3_, ammonia production from arginine; 15 °C/37 °C/45 °C, growth at each temperature; 6.5%, growth in 6.5% NaCl; SL, slime production from sucrose. +, positive reaction; −, negative reaction; (+), weak positive reaction; ++, strong positive reaction; V, variable reaction.

**Table 3:** Biochemical differentiation of 96 Anthotyros whey cheese isolates of the genus *Leuconostoc* in biotypes, their numerical distribution in each cheese batch and species identification of representative isolates of each biotype by 16S rRNA sequencing.

*Leuconostoc* Biotypes	SL	Acid Production from (Key Sugar Fermentation Reactions)						Whey Cheese Batch				Total	Representative Strains’ Codes	16S rRNA Identification
		LARA	GAL	LAC	RAF	TRE	DXYL	A	B	C	D		WM prefix	
L1	−	−	+	+	−	+	+	4	6	10	19	39	106, 109B, 123, 136, 137, 153	*Leuconostoc mesenteroides*
L2	−	+	+	+	+	+	+	10	9	5	1	25	105, 110A, 122A	*Leuconostoc mesenteroides*
L3	−	+	+	+	−	+	+	4	6	1	0	11	121	*Leuconostoc mesenteroides*
L4	+	15/17	+	+	15/17	+	+	11	4	2	0	17	107, 108	*Leuconostoc mesenteroides*
L5	−	−	+	+	+	−	−	0	0	2	2	4	118	*Leuconostoc lactis*
Total isolates								29	25	20	22	96		

SL, Slime production from sucrose; LARA, L-arabinose; GAL, Galactose; LAC, Lactose; RAF, Raffinose; TRE, Trehalose; DXYL, D-xylose. +, positive reaction; −, negative reaction; 15/17, 15 out of the 17 isolates were positive.

**Table 4:** Biochemical characterization of 13 Anthotyros whey cheese isolates of the genus *Carnobacterium* at the species and biotype level and their numerical distribution in whey each cheese batch.

Biochemical Test	*Carnobacterium* Biotypes			
	C1	C2	C3	C4
CO_2_ from glucose	(+)	−	−	−
Arginine hydrolysis	+	−/(+)	−/(+)	+
Esculin hydrolysis	+	+	+	+
Acid produced from:				
Amygdalin	+	+	+	+
Arabinose	−	−	−	−
Galactose	+/+d	(+)	(+)	+d
Gluconate	((+))	−	−	((+))
Glycerol	+	+	+	+
Inulin	−	−	−	−
Lactose	+	+	+	+
Mannitol	+/(+)	((+))	((+))	(+)
Melezitose	−	−	−	−
Melibiose	−	−	+	+
Methy-D-glucoside	+/+d	−	−	−
Ribose	+	+	+	+
Tagatose	−	−	−	−
Trehalose	+	−	−	+
Turanose	−	−	−	+
Xylose	−	−	−	+
Total isolates	8	2	1	2
Batch A	0	0	0	0
Batch B	5	0	0	0
Batch C	3	2	1	2
Batch D	0	0	0	0

+, positive reaction; −, negative reaction; (+) weak positive reaction; ((+)), very weak reaction; +d, delayed reaction. Reactions are tabulated according to Hammes and Hertel [50] for the species differentiation within the genus *Carnobacterium*. All *Carnobacterium* spp. in the *Bergey’s Manual* and all whey cheese biotypes of this study produce acid from cellobiose, fructose, glucose, maltose, mannose, salicin and sucrose but not from adonitol, dulcitol, glycogen, inositol, raffinose, rhamnose and sorbitol, as determined by their API50 CHL identification method.

**Table 5:** Numerical and percentage (%) distribution of the 120 identified LAB species isolates in the four Anthotyros whey cheese batches in association with the numerical distribution on their enumeration/isolation agar media.

Species	Whey Cheese Batch Isolates (%)				Total Isolates (%)	Enumeration/Isolation Agar Medium/ Incubation Temperature		
	A	B	C	D		MPCA/37 °C	MRS/30 °C	TSAYE/12 °C
*Leuconostoc mesenteroides*	29 (96.7)	25 (83.3)	18 (60.0)	20 (66.7)	92 (76.7)	28	39	25
*Leuconostoc lactis*			2 (6.7)	2 (6.7)	4 (3.3)	1		3
*Carnobacterium maltaromaticum*		5 (16.7)	8 (26.6)		13 (10.9)	4		9
*Streptococcus thermophilus*				7 (23.3)	7 (5.9)	7		
*Enterococcus faecium*				1 (3.3)	1 (0.8)		1	
*Lactococcus lactis*			2 (6.7)		2 (1.6)			2
Mesophilic *Lactobacillus* sp.	1 (3.3)				1 (0.8)			1
Total isolates	30	30	30	30	120	40	40	40

MPCA/37 °C, milk plate count agar incubated at 37 °C for 48 h; MRS/30 °C, de Man Rogosa Sharpe agar incubated at 30 °C for 72 h; TSAYE/12 °C, tryptic soy agar with 0.6% yeast extract incubated at 12 °C for up to 7 days.

**Table 6:** Identification of 74 representative Anthotyros whey cheese spoilage isolates of Gram-negative, oxidase-negative, fermentative bacteria recovered from the populations grown on CFC, MPCA and/or TSAYE plates in Table 1, as determined by the API 20E identification method.

Test	Reactions/Enzymes	*Hafnia* *alvei* I	*Hafnia* *alvei* II	*Serratia* *liquefaciens* I	*Serratia* *liquefaciens* II	*Rahnella* *Aquatilis*	*Pantoea* sp.	*Klebsiella* *oxytoca*	*Enterobacter/* *E. cloacae*
	Number of isolates ^1^	11 (30)	6 (11)	4 (4)	11 (13)	8 (8)	1 (1)	2 (3)	4 (4)
ONPG	Β-galactosidase	+	+	+	+	+	+	+	+
ADH	Arginine dihydrolase	−	−	−	−	−	−	−	+
LDC	Lysine decarboxylase	+	+	+	+	−	−	+	−
ODC	Ornithine decarboxylase	+	+	+	+	−	−	−	+
CIT	Citrate utilization	+	−	+	+	−	−	+	+
H_2_S	H_2_S production	−	−	−	−	−	−	−	−
URE	Urease	−	−	−	−	−	−	−/+	−
TDA	Tryptophane deaminase	−	−	−	−	−	−	−	−
IND	Indole production	−	−	−	−	−	−	+	−
VP	Acetoin production	−/+	−/+	+	+	+	+	+	+
GEL	Gelatinase	−	−	−	+	−	−	−	−
GLU	Glusose (F/O)	+	+	+	+	+	+	+	+
MAN	Mannitol (F/O)	+	+	+	+	+	+	+	+
INO	Inositol (F/O)	−	−	+	+	−	−	+	-
SOR	Sorbitol(F/O)	−	−	+	+	+/−	−	+	−/+
RHA	Rhamnose (F/O)	+	+	−	−	+	−	+	+
SAC	Saccharose (F/O)	−	−	+	+	+	+	+	+
MEL	Melibiose (F/O)	−	−	−	+	+	+	+	+
AMY	Amygdalin (F/O)	−	−	+	+	+	+	+	+
ARA	Arabinose (F/O)	+	+	+	+	+	−	+	+
OX	Oxidase reaction	−	−	−	−	−	−	−	−
	API code	5304112 5305112	5104112 5105112	5305723	5307763	1005573 1005173	1005161	5245773 5255773	3305173 3305573
	Identification accuracy	Excellent	Excellent	Very good	Very good	Low	Acceptable	Good	Excellent/Good

^1^ The first number indicates the isolates tested by the API 20E kit; the second number in bracket indicates the total number of isolates of each species recovered from the agar plates. +, positive colored reaction according to the API 20E manual instructions ; −, negative colored reaction according to the API 20E manual instructions.

**Table 7:** Identification of 22 representative Anthotyros whey cheese spoilage isolates of Gram-negative, oxidase-positive and/or non-fermentative bacteria recovered from the populations grown on CFC and/or TSAYE plates in Table 1, as determined by the API 20E identification method.

Test	Reactions/Enzymes	*Aeromonas* sp./ *A. salmonicida*	*Pseudomonas* sp. I	*Pseudomonas* sp. II	*Flavibacterium* *oryzihabitans*
	Number of isolates ^1^	4 (9)	4 (9)	2 (2)	1 (2)
ONPG	β-galactosidase	−	−	−	−
ADH	Arginine dihydrolase	−	+	+	−
LDC	Lysine decarboxylase	−	−	−	−
ODC	Ornithine decarboxylase	−	−	−	−
CIT	Citrate utilization	−	−/(+)	−	+
H_2_S	H_2_S production	−	−	−	−
URE	Urease	−	−	−	−
TDA	Tryptophane deaminase	−	−	−	−
IND	Indole production	−	−	−	−
VP	Acetoin production	−	+	−	−
GEL	Gelatinase	+	−	−	−
GLU	Glusose (F/O)	−/(+)	−/+	+	−
MAN	Mannitol (F/O)	−/(+)	−	−	−
INO	Inositol (F/O)	−	−	−	−
SOR	Sorbitol(F/O)	−	−	−	−
RHA	Rhamnose (F/O)	−	−	−	−
SAC	Saccharose (F/O)	−	−	−	−
MEL	Melibiose (F/O)	−	−	+	−
AMY	Amygdalin (F/O)	−	−	−	−
ARA	Arabinose (F/O)	−	−	+	−
OX	Oxidase reaction	+	+	+	−
	API code	0002004 0006104	2001004 2205004	2004046	0200000
	Identification accuracy	Low/Very good	Acceptable/Very good	Very good	Acceptable

^1^ The first number indicates the isolates tested by the API 20E kit; the second number in bracket indicates the total number of isolates of each species recovered from the agar plates. +, positive colored reaction according to the API 20E manual instructions ; −, negative colored reaction according to the API 20E manual instructions.

**Table 8:** Numerical distribution of the 96 isolates of Gram-negative spoilage bacteria in the four batches of Anthotyros whey cheese.

Genus/Species	Biotype	Whey Cheese Batch				Total Isolates
		A	B	C	D	
*Hafnia alvei*	I			30		30
*Hafnia alvei*	II				11	11
*Serratia liquefaciens*	I			4		4
*Serratia liquefaciens*	II	3	10			13
*Rahnellaaquatilis*		5	3			8
*Pantoea* sp.				1		
*Klebsiella oxytoca*				1	2	3
*Enterobacter* sp./*E. cloacae*				2	2	4
*Aeromonas* sp.					9	9
*Pseudomonas* sp.	I	5	4			9
*Pseudomonas* sp.	II	2				2
*Flavibacterium* sp.					2	2
Total isolates/batch		15	17	38	26	96

## Data Availability

The data presented in this study, as well as the bacterial isolates identified, are available on request from the corresponding author.

## Supplementary Materials

The following are available online at https://www.mdpi.com/article/10.3390/foods10122946/s1, Table S1: Changes in pH values of fresh, vacuum-packaged Anthotyros whey cheeses without (CN) or with (ENT) addition of 5% crude enterocin A-B-P extract during storage at 4 °C. Table S2: Growth of total mesophilic dairy bacteria (log CFU/g; enumerated on Milk Plate Count agar [MPCA] at 37 °C) during refrigerated (4 °C) storage of fresh, vacuum-packaged Anthotyros whey cheeses without (CN) or with (ENT) addition of 5% crude enterocin A-B-P extract. Table S3: Growth of total psychrotrophic bacteria (log CFU/g; enumerated on Tryptone Soya Agar with 0.6% Yeast Extract [TSAYE] at 12 °C) during refrigerated (4 °C) storage of fresh, vacuum-packaged Anthotyros whey cheeses without (CN) or with (ENT) addition of 5% crude enterocin A-B-P extract. Table S4: Growth of total lactic acid bacteria (LAB) (log CFU/g; enumerated on de Man, Rogosa, Sharpe [MRS] agar at 30 °C) during refrigerated (4 °C) storage of fresh, vacuum-packaged Anthotyros whey cheeses without (CN) or with (ENT) addition of 5% crude enterocin A-B-P extract. Table S5: Growth of *Pseudomonas*-like and related Gram-negative bacteria (log CFU/g; enumerated on Cetrimide-Fucidin-Cephaloridine [CFC] agar at 25 °C) during refrigerated (4 °C) storage of fresh, vacuum-packaged Anthotyros whey cheeses without (CN) or with (ENT) addition of 5% crude enterocin A-B-P extract.
