# Supplementary material for: Assessment of the Spoilage Microbiota during Refrigerated (4 °C) Vacuum-Packed Storage of Fresh Greek Anthotyros Whey Cheese without or with a Crude Enterocin A-B-P-Containing Extract
Source: Foods. 2021 Nov 30;10(12):2946. doi: 10.3390/foods10122946 (PMC8701269; doi:10.3390/foods10122946)
Supplement: Supplementary file 1 [file foods-10-02946-s001.zip › foods-1464957-supplementary.pdf]

Table S1. Changes in pH values of fresh, vacuum-packaged Anthotyros whey cheeses without (CN) or with (ENT) addition of 5% crude enterocin A-B-P extract during storage at 4°C

**Dairy Plant**	**Cheese batch**	**Treatment**	**Days of storage at 4oC**				
			**0**	**8**	**15**	**30**	**40**
**First dairy (Pappas Bros.)**	**A**	**CN**	6.93	6.86	6.38	5.54	5.15
		**ENT**	6.93	6.80	5.29	4.57	4.48
	**B**	**CN**	6.96	6.78	6.18	5.77	5.42
		**ENT**	6.96	6.80	5.84	4.75	4.69
**Second dairy**	**C**	**CN**	6.75	7.20	6.19	5.50	5.05
		**ENT**	6.70	7.12	6.53	5.22	4.89
	**D**	**CN**	6.57	6.52	6.08	5.22	4.93
		**ENT**	6.72	6.56	6.27	4.92	4.44
**Total mean (SD value)**	**A+B+C+D**	**CN**	6.80 (0.18)	6.84 (0.28)	6.21 (0.13)	5.51 (0.23)	5.14 (0.21)
		**ENT**	6.83 (0.14)	6.82 (0.23)	5.98 (0.54)	4.87 (0.28)	4.63 (0.21)

Table S2. Growth of total mesophilic dairy bacteria (log CFU/g; enumerated on Milk Plate Count agar [MPCA] at 37°C) during refrigerated (4°C) storage of fresh, vacuum-packaged Anthotyros whey cheeses without (CN) or with (ENT) addition of 5% crude enterocin A-B-P extract

**Dairy Plant**	**Cheese batch**	**Treatment**	**Days of storage at 4oC**				
			**0**	**8**	**15**	**30**	**40**
**First dairy (Pappas Bros.)**	**A**	**CN**	4.01	6.38	7.64	8.79	9.20
		**ENT**	4.01	6.52	9.20	9.23	8.76
	**B**	**CN**	3.45	6.12	6.85	8.35	8.78
		**ENT**	3.45	4.78	7.84	8.91	8.76
**Second dairy**	**C**	**CN**	4.14	7.29	8.42	8.62	8.79
		**ENT**	5.78	6.13	8.36	8.84	8.90
	**D**	**CN**	6.19	8.13	8.26	8.15	7.95
		**ENT**	6.32	7.72	8.06	8.26	8.23
**Total mean (SD value)**	**A+B+C+D**	**CN**	4.45 (1.20)	6.98 (0.92)	7.79 (0.71)	8.48 (0.28)	8.68 (0.52)
		**ENT**	4.89 (1.38)	6.29 (1.21)	8.37 (0.60)	8.81 (0.40)	8.66 (0.30)

Table S3. Growth of total psychrotrophic bacteria (log CFU/g; enumerated on Tryptone Soya Agar with 0.6% Yeast Extract [TSAYE] at 12 ^{o}C) during refrigerated (4°C) storage of fresh, vacuum-packaged Anthotyros whey cheeses without (CN) or with (ENT) addition of 5% crude enterocin A-B-P extract

Dairy Plant	Cheese batch	Treatment	Days of storage at 4oC				
			0	8	15	30	40
First dairy (Pappas Bros.)	A	CN	4.08	7.60	8.32	8.96	9.05
		ENT	4.28	7.22	9.31	9.23	8.88
	B	CN	3.61	6.79	9.21	8.94	9.17
		ENT	3.85	7.00	9.20	9.10	9.17
Second dairy	C	CN	2.30	6.91	8.41	8.94	9.27
		ENT	3.15	6.56	8.58	8.97	9.08
	D	CN	5.69	7.78	8.61	8.64	8.64
		ENT	5.71	8.01	8.62	8.91	9.04
Total mean (SD value)	A+B+C+D	CN	3.92 (1.40)	7.27 (0.49)	8.64 (0.40)	8.87 (0.15)	9.03 (0.28)
		ENT	4.25 (1.08)	7.20 (0.61)	8.93 (0.38)	9.05 (0.14)	9.04 (0.12)

Table S4. Growth of total lactic acid bacteria (LAB) (log CFU/g; enumerated on de Man, Rogosa, Sharpe [MRS] agar at 30°C) during refrigerated (4°C) storage of fresh, vacuum-packaged Anthotyros whey cheeses without (CN) or with (ENT) addition of 5% crude enterocin A-B-P extract

Dairy Plant	Cheese batch	Treatment	Days of storage at 4oC				
			0	8	15	30	40
First dairy (Pappas Bros.)	A	CN	<2.00	4.18	6.48	8.74	9.13
		ENT	<2.00	3.95	6.60	9.21	8.94
	B	CN	2.30	5.16	6.81	8.46	8.99
		ENT	<2.00	5.23	6.78	8.91	8.75
Second dairy	C	CN	<2.00	5.83	7.11	8.02	8.66
		ENT	<2.00	5.53	7.95	8.83	8.86
	D	CN	4.24	7.10	8.43	8.72	8.65
		ENT	4.30	7.24	8.36	8.81	9.02
Total mean (SD value)	A+B+C+D	CN	2.64 (1.08)	5.57 (1.23)	7.21 (0.85)	8.49 (0.34)	8.86 (0.24)
		ENT	2.58 (1.15)	5.49 (1.35)	7.42 (0.86)	8.94 (0.19)	8.89 (0.12)

Table S5. Growth of *Pseudomonas*-like and related gram-negative bacteria (log CFU/g; enumerated on Cetrimide-Fucidin-Cephaloridine [CFC] agar at 25°C) during refrigerated (4°C) storage of fresh, vacuum-packaged Anthotyros whey cheeses without (CN) or with (ENT) addition of 5% crude enterocin A-B-P extract

**Dairy Plant**	**Cheese batch**	**Treatment**	**Days of storage at 4oC**				
			**0**	**8**	**15**	**30**	**40**
**First dairy (Pappas Bros.)**	**A**	**CN**	4.47	6.21	8.29	8.27	6.36
		**ENT**	4.47	6.28	7.95	6.63	5.31
	**B**	**CN**	4.44	6.47	7.91	8.22	7.72
		**ENT**	4.44	6.45	8.14	7.30	5.72
**Second dairy**	**C**	**CN**	<2.00	5.48	7.89	7.94	7.76
		**ENT**	<2.00	5.29	7.68	8.02	8.01
	**D**	**CN**	2.48	7.31	7.28	5.20	4.88
		**ENT**	2.70	7.10	7.39	6.89	<3.00
**Total mean (SD value)**	**A+B+C+D**	**CN**	3.35 (1.29)	6.37 (0.76)	7.84 (0.42)	7.41 (1.48)	6.68 (1.37)
		**ENT**	3.40 (1.25)	6.28 (0.75)	7.79 (0.33)	7.21 (0.61)	5.51 (2.05)
